# Adverse Events Associated with the Clinical Use of Bee Venom: A Review

**DOI:** 10.3390/toxins14080562

**Published:** 2022-08-18

**Authors:** Jaehee Yoo, Gihyun Lee

**Affiliations:** 1 Department of Acupuncture and Moxibustion Medicine, Dongshin University, 67 Dongshindae-gil, Naju 58245, Korea; 2 College of Korean Medicine, Dongshin University, 67 Donshindae-gil, Naju 58245, Korea

**Keywords:** bee venom, bee venom acupuncture, adverse events, adverse effect

## Abstract

Bee venom is used to treat various diseases but can cause a tickling sensation and anaphylaxis during clinical treatment. Adverse events (AEs) associated with bee venom may vary depending on the dosage, method, route of administration, and the country, region, and user. We summarized the AEs of bee venom used in various ways, such as by the injection of extracts, venom immunotherapy (VIT), live bee stings, or external preparations. We conducted a search in eight databases up to 28 February 2022. It took one month to set the topic and about 2 weeks to set the search terms and the search formula. We conducted a search in advance on 21 February to see if there were omissions in the search terms and whether the search formula was correct. There were no restrictions on the language or bee venom method used and diseases treated. However, natural stings that were not used for treatment were excluded. A total of 105 studies were selected, of which 67, 26, 8, and 4 were on the injection of extracts, VIT, live bee stings, and external preparation, respectively. Sixty-three studies accurately described AEs, while 42 did not report AEs. Thirty-five randomized controlled trials (RCTs) were evaluated for the risk of bias, and most of the studies had low significance. A large-scale clinical RCT that evaluates results based on objective criteria is needed. Strict criteria are needed for the reporting of AEs associated with bee venom

## 1. Introduction

Bee venom treatment uses the pharmacological effect of bee sting toxins and is widely used worldwide [1]. In addition to musculoskeletal diseases, bee venom is used for therapeutic purposes such as for uterine ovarian disease [2], cancer [3], and atopic dermatitis [4].

Bee venom treatment is performed in various ways, such as through apitoxin, bee venom acupuncture, venom immunotherapy (VIT), and live bee stings [5]. Among studies on the adverse events of bee venom, studies summarizing adverse events according to the type of paper have been conducted along with randomized controlled trials (RCTs) [6]. However, no studies have reported the side effects of bee venom treatment.

The toxin component of bee venom is presented to T cells by antigen-presenting cells in the skin and eventually causes an allergic reaction by producing IgE [7]. The most serious adverse event of bee venom treatment is anaphylaxis; however, the incidence is not high [8]. If anaphylaxis occurs, epinephrine may be treated preferentially [9]. However, owing to practical and ethical issues, strong evidence on the diagnosis and management of anaphylaxis is lacking [10]. Anaphylaxis can present similarly to acute asthma, local angioedema, fainting, and anxiety/panic seizures [11].

Treatment with bee venom can often cause adverse events by generating IgE [2], and it can appear in different ways depending on how the bee venom is stimulated. We aimed to investigate which method could safely use bee venom by classifying the adverse events during clinical use. Our results will help clinical therapists using bee venom to choose the method of bee venom stimulation and prepare for adverse events.

## 2. Results

### 2.1. Descriptions of Trials

A total of 1410 papers were searched using PubMed (226 papers), Cochrane (7), EMBASE (420), CINAHL (40), CNKI (296), NDSL (261), OASIS (21), KISS (37), KoreaMED (16), and KMBASE (84). After the exclusion of papers that did not meet the extraction conditions, 105 papers were finally selected. Bee venom stimulation methods included extract injections (67 studies) [12,13,14,15,16,17,18,19,20,21,22,23,24,25,26,27,28,29,30,31,32,33,34,35,36,37,38,39,40,41,42,43,44,45,46,47,48,49,50,51,52,53,54,55,56,57,58,59,60,61,62,63,64,65,66,67,68,69,70,71,72,73,74,75,76,77,78], venom immunotherapy (VIT; 26 studies) [79,80,81,82,83,84,85,86,87,88,89,90,91,92,93,94,95,96,97,98,99,100,101,102,103,104], live bee stings (8 studies) [105,106,107,108,109,110,111,112], and external preparations (4 studies) [113,114,115,116].

Forty-nine, twenty-eight, six, five, four, three, two, two, two, one, one, and one studies were conducted in China, Korea, Germany, Australia, Poland, Turkey, the United States, Spain, the Czech Republic, Greece, Belgium, and France, respectively. There were 33 case reports (CRs), 15 case series (CS), 14 cohort studies, 6 non-randomized controlled trials (nRCTs), and 37 RCTs. In the case of VIT, the purpose of treatment was to lower hypersensitivity to venom, and the diseases to which treatment was applied were noticeably more musculoskeletal diseases such as rheumatoid arthritis, ankylosing spondylitis, osteoarthritis, frozen shoulder, and lumbar disc herniation. In addition, neuropathy, urticaria, tonsillitis, rhinitis, acne, facial palsy, and menstrual pain were treated.

The venom type mainly used was bee venom, but 19 studies used wasp venom in VIT. In most studies, the results of the pre-skin test were not confirmed. In the case of extract injections, acupuncture, cupping, herbal medicine, acupotomy, moxibustion, and physical therapy were accompanied by bee venom treatment. In addition, drugs such as methotrexate, prednisolone acetate, seraxib capsules, and tramadol were identified. In the case of VIT, omalizumab was used when adverse events were severe during VIT rather than as a concomitant treatment. In the case of live bee stings, McKenzie’s methods, medication, and fluid were accompanied by the treatment. For external preparations, CO_2_ lasers and medication were used. Only 27 studies specified the capacity of bee venom.

### 2.2. Adverse Events

**Figure 1:**
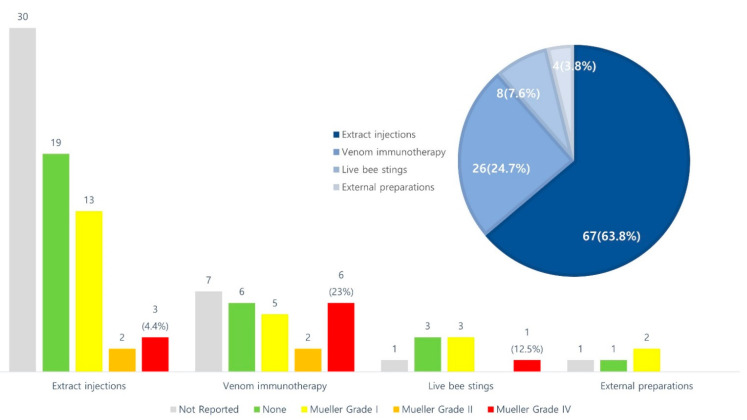
Adverse events summary. The contents related to the reporting of adverse events of each stimulation type of procedure are described. There are 67 extract injections, 26 venom immunotherapy, 8 live bee stings, and 4 external preparations. It was classified into not reported, none, and Mueller grades, and if several types of Mueller grades occurred, it was classified as a high grade. Mueller grade III was not reported in the selected studies.

The contents related to the reporting of adverse events are shown in Figure 1. The details are listed in Table 1, Table 2, Table 3 and Table 4. Twenty-eight studies reported no adverse events, thirty-four studies specifically reported adverse events, and forty-three studies did not include adverse events. In one CR, no adverse events were reported. Seven of the forty-three studies reported the occurrence of adverse events using the terms “skin problem” or “systemic reaction,” without describing the specific symptoms. As a result of confirming the severity of adverse events through Spilker’s classification (Table 5), there were 26 mild, 4 moderate, and 11 severe adverse events. According to Mueller grading (Table 6), there were 23 grade I, 4 grade II, 0 grade III, and 4 grade IV cases (Figure 1).

### 2.3. Risk of Bias in Included Studies

Among the 37 RCTs, 1 study that used bee venom for the intervention group and wasp venom for the control group and 1 study that used different doses of bee venom for the intervention and control groups were excluded. For the remaining 35 RCTs, the interventions, control group treatment contents, evaluation index, results, and effective values were summarized. Subsequently, the risk of bias (RoB) was evaluated based on the content of the included studies.

**Figure 2:**
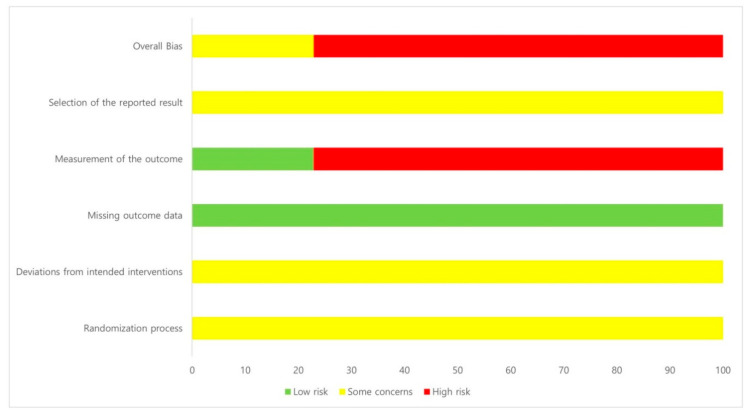
Risk of bias graph. Domain 1 (randomization process): random allocation and double blindness. Domain 2 (deviations from intended interventions): dropouts, insufficient sample size, and caregiver blindness. Domain 3 (missing outcome data): availability of results to all participants. Domain 4 (measurement of the outcome): appropriation or diversity of measurement methods. Domain 5 (selection of the reported result): this is performed by the implementation plan or protocol. Domain 6 (overall bias): combination of the five domains.

**Figure 3:**
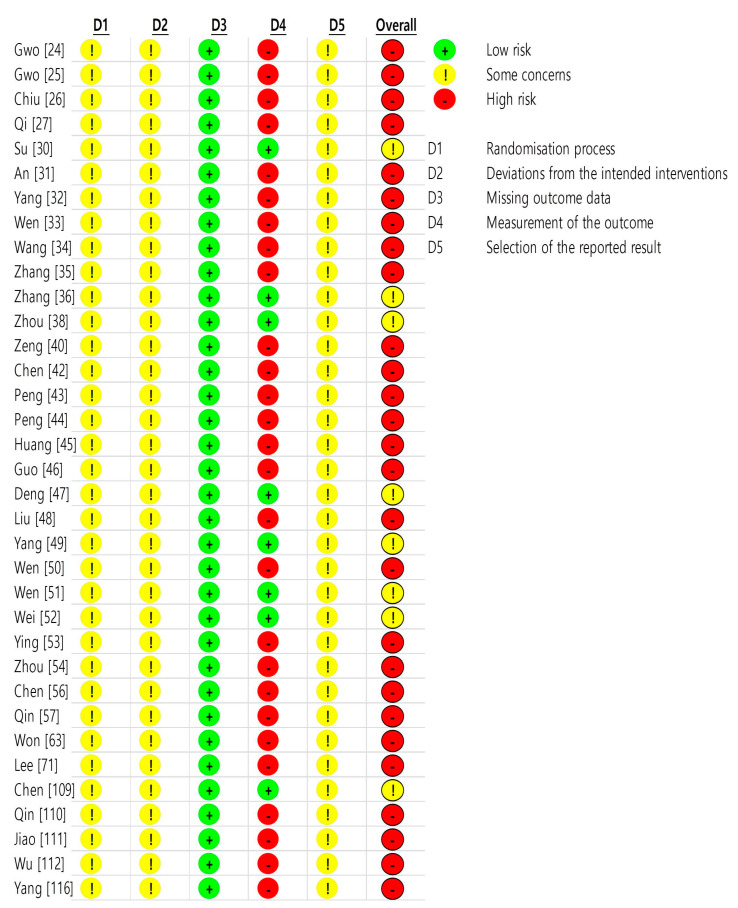
Risk of bias summary Domain 1 (randomization process): random allocation and double blindness. Domain 2 (deviations from intended interventions): dropouts, insufficient sample size, and caregiver blindness. Domain 3 (missing outcome data): availability of results to all participants. Domain 4 (measurement of the outcome): appropriation or diversity of measurement methods. Domain 5 (selection of the reported result): this is performed by the implementation plan or protocol. Domain 6 (overall bias): combination of the five domains.

All 35 studies in the first domain of random allocation and double blindness were evaluated as “some concerns.” In all studies, participants were randomly assigned. However, there was no information on blinding after the random assignment. All 35 studies were evaluated as “some concerns” in the second domain because there were dropouts, the sample size was not sufficient, or the caregiver was not blinded to the group assignment of the participants. In all 35 studies, the results of the study participants were evaluated as “low risk” because they appeared to be universally available to all participants. In the fourth domain, 8 studies were “low risk” because there was an objective outcome measurement method, but 27 studies were “high risk” because only scales based on the subjective symptoms of participants were used. All 35 studies were evaluated as having “some concerns” because no implementation plan or protocol was mentioned. The details are presented in Table 7 and Figure 2 and Figure 3.

## 3. Discussion

We conducted a literature search using eight databases: PubMed, Cochrane, EMBASE, CINAHL, CNKI, NDSL, OASIS, KISS, KoreaMED, and KMBASE. However, there were many cases in which access to Chinese-based databases was not possible, so an additional literature search could not be performed. Ultimately, 105 studies were included. There were forty-nine, twenty-eight, six, five, four, three, two, two, one, one, one, and one studies from China, Korea, Germany, Australia, Poland, Turkey, Spain, Czech Republic, Greece, Belgium, France, and Japan, respectively. As for the paper type, there were 37 RCTs, 33 CRs, 15 CSs, 14 cohort studies, and 6 nRCTs.

When classified according to the stimulation method of bee venom, there were 67, 26, 8, and 4 studies on extract injections, VIT, live bee stings, and external preparations, respectively. Twenty-seven studies described the injection capacity of bee venom, but few studies specifically described the dose that was injected into how many acupoints.

Twenty-eight studies reported no adverse events, thirty-four specifically reported adverse events, and the remaining forty-three studies partially or failed to describe adverse events. Seven of the forty-six studies did not describe specific symptoms of adverse events but described adverse events such as “skin problem” and “systemic reaction”. Based on Mueller’s classification, twenty-nine cases were grade I and two cases were grade II with the patients complaining of abdominal pain, chest pain, and vomiting. There was also one case of grade III, with the patient presenting with weakness and dyspnea, and eleven cases of grade IV, with patients suffering from hypotension and cyanosis. According to Spilker’s classification, 26 cases were “mild” with no functional disruption to daily activities, 4 cases were “moderate” with symptoms disappearing over time when additional treatment was applied, and 11 cases were “severe” with immediate treatment required or after-effects. Regarding “mild” symptoms, there were cases where it was accompanied by “moderate” to “severe” symptoms.

Bee venom injections are performed using refined bee venom. In this process, active ingredients can be extracted separately and allergens can be removed. Moreover, the capacity and concentration of the bee venom injections can be easily controlled [117]. Depending on the venom to be purified, snakes [118] and jellyfish [119] can be used instead of bees. However, as an invasive treatment, there may be a risk of infection, depending on the injection site. In addition, since the unification of terms, such as bee venom acupuncture and bee venom pharmacopuncture, has not been achieved, it is necessary to establish appropriate terminology.

VIT is a prophylactic method that aims to reduce hypersensitivity in individuals with hypersensitivity to venom [120]. If adverse events occur during follow-up, additional treatment such as the oral administration of omalizumab, an anti-IgE, may be introduced [121]. However, in the case of VIT, since it is targeted at people who have already experienced adverse events or hypersensitivity, it seems that the definition of an adverse event should be different.

Live bee stings may have similar effects to bee venom injections but are clinically impractical because they require live bees [122]. Bees vary slightly in composition and concentration, depending on the type and growth area [123]. In addition, live-bee dermatitis may occur if infected [124]. Since this method directly uses bees to sting, criteria that detail the infection control process, effective bee type, recommended time, and/or the number of stings are needed.

Bee venom is sometimes used as an external preparation, and honey, royal jelly, and bee venom are used to treat and prevent oral diseases [125], while cream containing bee venom is used to improve wrinkles [126]. A direct correlation between bee venom allergy and bee products has not been revealed, but some people are allergic to honey or propolis [127]. In the case of external preparations, more clinical studies are needed to determine the correlation between the concentration of ingredients, the amount of application, and allergies.

Of the 105 studies included in this review, only 63 reported specifically on the adverse events that occurred. VIT seems to be used in many Western countries, whereas bee venom injections and live bee stings seem to be used in many Eastern countries. Since the method of bee venom stimulation differs by country and culture, it is thought that the reporting method for the adverse events that occur may be different. There are criteria such as Mueller’s and Spilker’s classification, but these criteria do not appear to be essential in the reporting of venom treatment. Since bee venom has the potential to cause anaphylaxis, reports of side effects must be included in venom clinical trials.

RoB evaluation was conducted on 35 RCT studies. Each domain explains a randomization process, deviations from intended interventions, missing outcome data, a measurement of the outcome, selection of the reported result and overall bias. As shown in the RoB results, there were no studies with a low RoB. To supplement this study, objective and diverse scales are required in large RCTs to evaluate the effectiveness of bee venom, and a rigorous reporting framework for adverse events should be presented.

From the 105 studies reviewed, there were 10 studies in which Mueller’s grade IV adverse events occurred (3 extract injection studies, 6 VIT studies, and 1 live bee sting study). Only 2 studies were conducted in advance. The most serious adverse events that can occur with bee venom treatment were anaphylaxis and unrecoverable sequelae. Since there is a possibility of anaphylaxis, it is recommended that a person with medical knowledge manages patients undergoing a bee venom procedure. Further research is needed on the relationship between skin test results and serious adverse events. However, to reduce the occurrence of serious adverse events in clinical practice, skin tests should be conducted prior to treatment. In addition, since skin tests are used to adjust the concentration and capacity of the active ingredient, the live bee sting type is not recommended. In the selected papers, the capacity of bee venom was expressed in various ways, such as mL and cc. When researchers write papers or conduct experiments, it is necessary to use general units such as mg/kg, or to specify capacity units and concentrations of effective ingredients according to the purpose of the study. This study focuses on the adverse events of bee venom. If an additional comparative study on the effect, adverse events rate, and fatality rate according to the stimulation type is conducted, the clinician may use bee venom in consideration of the effect and adverse events.

## 4. Conclusions

This study reviewed the adverse effects of bee venom stimulation. Most of the RoB evaluations of RCT studies were not significant, and large-scale RCT studies with a system for reporting adverse events of bee venom are required. A skin test is needed to reduce the occurrence of adverse events, and a person who can cope with anaphylaxis should perform a bee venom procedure. It was confirmed that many studies omitted reports of adverse events. In order to analyze the occurrence and fatality rate of adverse events according to the stimulation type, it is essential to include a report of adverse events when using bee venom.

## 5. Methods

### 5.1. Search Method for Identifying Studies

This study included eight databases: PubMed(National Center for Biotechnology Information, Bethesda, Maryland, U.S.A.), Cochrane(John Wiley&Sons, Inc., London, UK, 2000), CINAHL(EBSCO Industries, Birmingham, AL, USA), CNKI(Tongfang Knowledge Network Technology Co., Ltd., Beijing, China, 2014), NDSL(Korea Institute of Science and Information Technology, Daejeon, Korea), OASIS(Korea Institute of oriental medicine, Daejeon, Korea, 2016), KISS(Korea Studies Information Co., Ltd., Paju, Gyeonggi-do, Korea), KoreaMED(Korea Association of Medical Journal Editors, Seoul, Korea), and KMBASE(MedRIC, Cheongju, Chungcheongbuk-do, Korea, 2000). The search was conducted using “bee venom acupuncture” and “adverse events” as keywords. There were no restrictions on the country or the language of the issue. The search was conducted up to 28 February 2022.

### 5.2. Inclusion Criteria

#### 5.2.1. Types of Studies

CRs, CSs, and nRCTs were included. Experimental, animal, and protocol studies were excluded.

#### 5.2.2. Types of Participants

There were no special restrictions on the diseases treated and patient characteristics.

#### 5.2.3. Types of Interventions

All treatments using bee venom were included in the intervention group. Non-intervention cases were excluded even if bee venom was used. In the case of RCTs, group classification according to the capacity of bee venom was included. Studies that included individuals who were accidentally stung by a bee (i.e., the sting was not part of their treatment) were excluded. There were no restrictions on the comparison group.

#### 5.2.4. Types of Outcome Measures

Contents related to adverse events were also extracted. The symptoms were classified into skin problems, systemic reactions, and others. The severity of the symptoms was classified as mild, moderate, and severe according to Spilker’s classification (Table 5) [128] and grades I to IV according to Mueller’s classification (Table 6) [129]. Causality was classified as certain, probable, possible, unlikely, unclassified, and unclassifiable according to the WHO-UMC causality scale (Table 8) [130]. No adverse events were described as “non-reported,” and no adverse events were “none”.

### 5.3. Data Selection and Extraction

#### 5.3.1. Selection of Studies

**Figure 4:**
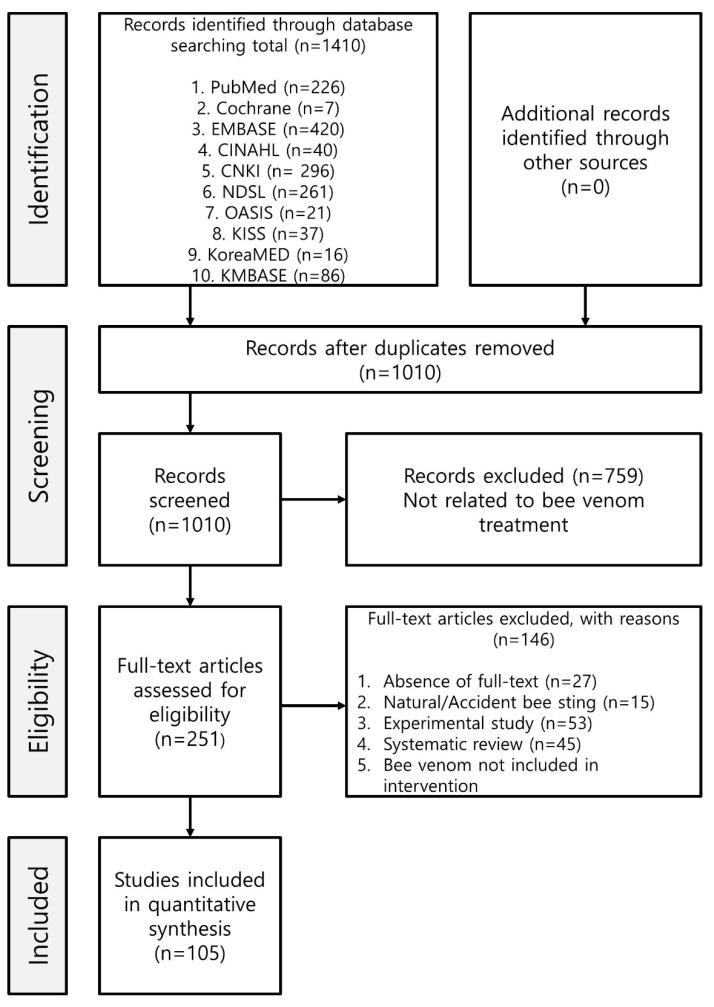
PRISMA flow diagram of the study selection. EMBASE: Excerpta Medica database; CINAHL: Cumulative Index to Nursing and Allied Health Literature; CNKI: China National Knowledge Infrastructure; NDSL: National Discovery for Science Library; OASIS: Oriental Medicine Advanced Searching Integrated System; KISS: Korean Studies Information Service System; KMBASE: Korea Medical database.

Two authors (JY and GL) independently searched each of the eight databases based on the abstracts. The full text was checked for papers for which the abstract was insufficient. The entire process was summarized according to the Preferred Reporting Items for Systematic Reviews and Meta-Analyses (PRISMA) guidelines (Figure 4) [131].

The study selection process is summarized in Figure 4. Duplicate studies and those that did not meet the selection criteria were excluded.

#### 5.3.2. Data Extraction

One author (JY) extracted the data, and the other (GL) inspected the extracted data. The number of participants, type of bee venom treatment method, outcomes, and the information related to adverse events were recorded.

#### 5.3.3. Assessment RCTs

Two reviewers evaluated the bias of RCT studies using the RoB evaluation [132]. The bias evaluation item consisted of five categories: (1) randomization process, (2) deviations from intended interventions, (3) missing outcome data, (4) measurement of the outcome, and (5) selection of the reported result. The first domain is whether random assignments and double blindness are properly performed, and the second domain is whether the dropout rate is high, the sample size is sufficient, or the caregiver is aware of the group assignment of the participants. The third domain concerned whether the results of the study were all available to the study participants. The fourth domain relates to whether the method of measuring results is the same and appropriate between groups, and the fifth domain relates to whether the research results were conducted using a pre-protocol. In addition, overall bias was evaluated by synthesizing five evaluation items. In each item, if there is no RoB, it is marked as “low risk”, if the RoB was high as “high risk”, and if there is no information on the item, it was marked as “some concerns”.

## Figures and Tables

**Table 1:** Basic characteristics of extract injection type.

First Author	Country	Reason	Paper Type	Number of Cases	Venom Type	Skin Test	Injection Amount	Concomitant Treatment	Adverse Events Symptoms	Adverse Events Severity	Adverse Events Type	Mueller Classification	Causality
Han [12]	Korea	pain prevention therapy	CR	1	bees	NR	NR	NR	skin atrophy	severe	SP	Gr1	probable
Castro [13]	U.S.A.	multiple sclerosis	CR	9	bees	NR	NR	NR	none	-	-	-	-
Lee [14]	Korea	facial palsy	cohort	108	bees	tested A: negative B: positive	0.1–0.2 mL	-	rash pruritus swelling vesicles erythema hives	mild	SP	Gr 1	probable
Jeong [15]	Korea	rotator cuff disease	cohort	4	bees	tested (negative)	0.1~0.5 cc	acupuncture herbal medicine physical therapy	none	-	-	-	-
Kim [16]	Korea	CRPS	CR	1	bees	NR	0.15–0.4 mL	anticonvulsant tricyclic antidepressant analgesic	hypersensitivity dyspepsia rash depression	mild	SP SR	Gr1	possible
Kim [17]	Korea	allergic rhinitis	CR	2	bees	NR	0.1~0.3 cc	acupuncture	none	-	-	-	-
Moon [18]	Korea	Fibromyalgia	CR	1	bees	NR	0.25 ccx4	acupuncture pharmacopuncture (hwangryunhaedok-tang) cupping moxibustion herbal medicine	None	-	-	-	-
Park [19]	Korea	lumbar disc herniation	cohort	A:12 B:10	A:- B:bees	tested (negative)	A:1.0 cc B:1.0 cc	A: Shinbaro, acupuncture, cupping, moxibustion, herbal medicine, physical therapy B: acupuncture, cupping, moxibustion, herbal medicine, physical therapy	redness itching	mild	SP	Gr1	possible
An [20]	Korea	Systemic Lupus Erythematosus	CR	1	bees	NR	NR	pharmacopuncture acupuncture herbal medicine	None	-	-	-	-
Kim [21]	Korea	survey study	cohort	A:132 B:336	A:bees B:-	tested (negative)	NR	A:- B:NR	point pain redness swelling numbness	mild	SP	Gr1	possible
Lee [22]	Korea	refractory postherpetic neuralgia	CR	1	Bees	tested (negative)	NR	NR	none	-	-	-	-
Kam [23]	China	lung cancer	nRCT	A:85 B:82	A:bees B:-	NR	NR	A:- B: granulocyte colony-stimulating factor	NR	NR	NR	NR	NR
Gwo [24]	China	chronic urticaria	RCT	A:50 B:50	A:bees B:-	NR	NR	A: herbal medicine B: acupuncture, herbal medicine	NR	mild	NR	NR	NR
Gwo [25]	China	ankylosing	RCT	A:30 B:30	A:bees	NR	NR	A: Bee’s oral medicine B: western medicine	NR	NR	NR	NR	NR
Chiu [26]	China	rheumatoid arthritis	RCT	A:35 B:35	A:bees B:-	NR	NR	A: methotrexine B: methotrexine, prednisolone acetate	NR	NR	NR	NR	NR
Qi [27]	China	rheumatoid arthritis	RCT	A:49 B:49	A:bees B:-	NR	NR	A: NR B: western medicine	NR	NR	NR	NR	NR
She [28]	China	ankylosing	nRCT	A:68 B:38	A:bees B:-	NR	NR	A: chuna B: oral seraxib capsules	stomachache	mild	SR	Gr2	possible
Su [29]	China	ankylosing	CR	NR	bees	NR	NR	NR	none	-	-	-	-
Su [30]	China	enlargement of mammary gland	RCT	A:30 B:30 C:30	A:bees B:bees C:-	NR	NR	A:- B: acupuncture C: acupuncture	fever urticaria lymphoma cirrhosis bleeding	moderate	SP SR	Gr1	probable
An [31]	China	cancerous pain from lung cancer	RCT	A:39 B:39	A:bees B:-	NR	NR	A: hydroxycodone tablets B: hydroxycodone tablets	NR	NR	NR	NR	NR
Yang [32]	China	rheumatoid arthritis	RCT	A:46 B:46	A:bees B:-	NR	NR	A: Chinese medicine B: routine treatment	NR	NR	NR	NR	NR
Wen [33]	China	ankylosing	RCT	A:40 B:40	A:bees B:-	NR	NR	A:- B: sulfasalazine, diclofenac	NR	NR	NR	NR	NR
Wang [34]	China	cancer pain	RCT	A:44 B:43	A:bees B:-	NR	NR	A: fentanyl percutaneous patch B: fentanyl percutaneous patch	NR	NR	NR	NR	NR
Zhang [35]	China	frozen shoulder	RCT	A:33 B:32 B:32	A:bees B:- C:-	NR	NR	A: acupotomy B: acupotomy, triamcinolone acetonide C: acupotomy	NR	NR	NR	NR	NR
Zhang [36]	China	facial palsy	RCT	A:36 B:35	A:bees B:-	NR	NR	A: acupuncture B: acupuncture	redness itching	mild	SP	Gr1	possible
Zhou [37]	China	ankylosing	CS	40	bees	NR	NR	Chinese medicine	NR	NR	NR	NR	NR
Zhou [38]	China	rheumatoid arthritis	RCT	A:40 B:30 C:30	A:bees	NR	NR	A:- B: electro acupuncture C: western medicine	None	-	-	-	-
Zhu [39]	China	ankylosing	CS	56	bees	tested (negative)	NR	Chinese medicine	fever itching urticaria pain anaphylaxis	severe	SP SR	Gr4	probable
Zeng [40]	China	ankylosing	RCT	A:54 B:54	A:bees B:-	NR	NR	A: moxibustion B: acupuncture	NR	NR	NR	NR	NR
Chen [41]	China	leukocyte reduction after colorectal cancer chemotherapy	nRCT	A:33 B:33	A:bees B:-	NR	NR	A:- B: white elm tablets	fever	mild	SP	Gr1	possible
Chen [42]	China	rheumatoid arthritis	RCT	A:30 B:30	A:bees B:-	NR	NR	A:- B: oral methotrexate, celecoxib	none	-	-	-	-
Peng [43]	China	cancer pain	RCT	A:31 B:33	A:bees B:	NR	NR	A: tramadol 100 mg B: tramadol 100 mg	NR	NR	NR	NR	NR
Peng [44]	China	cancer pain	RCT	A:30 B:30	A:bees B:-	NR	NR	A: pain medicine 3rd phase B: pain medicine 3rd phase (WHO recommended)	NR	NR	NR	NR	NR
Huang [45]	China	rheumatoid arthritis	RCT	A:30 B:30	A:bees B:-	NR	NR	A:- B: hemp tablet	NR	NR	NR	NR	NR
Guo [46]	China	ankylosing	RCT	A:36 B:36	A:bees B:-	NR	NR	A:- B: western treatment	NR	NR	NR	NR	NR
Deng [47]	China	RA	RCT	A:20 B:20 C:20	A:bees B:- C:-	NR	NR	A: metrotrexate B: metrotrexate C: strong metrotrexate	NR	NR	NR	NR	NR
Liu [48]	China	RA	RCT	A:50 B:50	A:bees B:-	NR	NR	A: western medicine B: western medicine	NR	NR	NR	NR	NR
Yang [49]	China	diabetic neuropathy	RCT	A:25 B:25	A:bees B:-	NR	NR	A: epalrestat, methylcobalamin B: epalrestat, methylcobalamin	none	-	-	-	-
Wen [50]	China	postpartum pain	RCT	A:41 B:40	A:bees B:-	NR	NR	A: herbal fumigation B: diclofenac natrium minidose	NR	NR	NR	NR	NR
Wen [51]	China	postherpetic neuralgia	RCT	A:36 B:36	A:bees B:-	NR	NR	A:- B: unknown injection	NR	NR	NR	NR	NR
Wei [52]	China	rheumatoid arthritis	RCT	A:30 B:30	A:bees B:-	NR	NR	A:- B: Chinese medicine	NR	NR	NR	NR	NR
Ying [53]	China	shoulder pain	RCT	A:60 B:60	A:bees B:-	NR	NR	A:- B: massage, acupuncture	NR	NR	NR	NR	NR
Zhou [54]	China	neurotic tinnitus	RCT	A:30 B:30	A:bees B:-	NR	NR	A: heating needle B: flunarizine hydrochloride capsule, mecobalamin minidose	NR	NR	NR	NR	NR
Chen [55]	China	lumbar disc herniation	CS	4000	bees	NR	NR	chuna	NR	NR	NR	NR	NR
Chen [56]	China	rheumatoid arthritis	RCT	A:30 B:30 C:30	A:bees (high) B:bees (low) C:-	NR	NR	A:- B:- C: methotrexate 10 mg, cerecoxib 0.2 g	NR	NR	NR	NR	NR
Qin [57]	China	rheumatoid arthritis	RCT	A:32 B:28	A:bees B:-	NR	NR	A: xianlong granule B: methotrexate	NR	NR	NR	NR	NR
Han [58]	China	diabetes	CS	80	bees	NR	NR	Chinese medicine	NR	NR	NR	NR	NR
Kim [59]	Korea	NR	CR	1	bees	NR	NR	NR	papules crust	moderate	SP	Gr1	probable
Jeong [60]	Korea	NR	CR	1	bees	NR	NR	NR	mycobacterium massiliense granulomatous	moderate	SP	Gr1	probable
Lee [61]	Korea	NR	cohort	8580	bees	NR	NR	NR	anaphylaxis shock	severe	SR	Gr4	probable
Yook [62]	Korea	effect	nRCT	A:19 B:23	A:bees B:-	NR	0.05x4	A:- B: normal saline	Body ache itching sense redness swelling headache dizziness fatigue nausea	mild	SP SR	Gr2	possible
Won [63]	Korea	osteoarthritis	RCT	A:25 B:26 C:26 D:24	A,B,C:bees D:-	NR	A:~0.7 mg B:~1.5 mg C:~2.0 mg D:1000 mg	A,B,C:- D: nabumetone	Itching body ache	mild	SP SR	Gr1	possible
Kim [64]	Korea	lower urinary tract symptoms	CS	41	bees	NR	NR	NR	none	-	-	-	-
Kim [65]	Korea	NR	CR	2	bees	NR	NR	NR	(1) hypotension, drowsy mentality, dyspnea, vomiting (2) itching sensation, urticaria, breathlessness, abdominal pain	severe	SP SR	(1) Gr4 (2) Gr3	probable
Li [66]	China	rheumatoid arthritis	CS	225	bees	NR	NR	NR	NR	NR	NR	NR	NR
Ma [67]	China	cancer pain	CR	NR	bees	NR	0.5 mg	morphine sulfate	constipation drowsy	mild	SPSR	Gr1	possible
Yeon [68]	Korea	low back pain	CR	2	bees	tested (negative)	0.2 cc	(1) fire needling (2) -	NR	NR	NR	NR	NR
Lee [69]	Korea	trigger finger	CR	1	bees	tested (negative)	0.3 cc	NR	none	-	-	-	-
Hwang [70]	Korea	systemic sclerosis	CR	1	bees	tested (negative)	NR	NR	none	-	-	-	-
Lee [71]	Korea	non-specific neck pain	RCT	A:30 B:30	A:bees B:-	NR	A:NR B:180 mg	A:- B: loxoprofen	none	-	-	-	-
Kim [72]	Korea	knee OA	nRCT	A:40 B:NR	A:bees B:-	NR	NR	A:- B: acupuncture	NR	NR	NR	NR	NR
Han [73]	Korea	OA with DM	CR	1	bees	NR	NR	herbal medicine physical therapy acupuncture	none	-	-	-	-
Lee [74]	Korea	lower back pain	cohort	523	bees	NR	0.1–1.2 mL	NR	local hypersensitivity	moderate	SP	Gr1	possible
Lee [75]	Korea	Raynaud’s disease	CR	1	bees	NR	NR	herbal medicine (Gamiguibi-tang)	none	-	-	-	-
Park [76]	Korea	chemotherapy-induced peripheral neuropathy	CR	5	bees	tested (negative)	NR	NR	none	-	-	-	-
Bong [77]	Korea	acute low back pain	CR	3	bees	NR	NR	acupuncture cupping herbal medicine physical therapy	none	-	-	-	-
Jo [78]	Korea	periungual warts	CR	11	bees	NR	NR	acupuncture herbal medicine moxibustion	none	-	-	-	-

NR: not reported; CR: case report; CS: case series; nRCT: non-randomized controlled trial; RCT: randomized controlled trial; SP: skin problem; SR: systemic reaction; CRPS: complex regional pain syndrome; Adverse events severity: Spilker’s classification Section 5.2.4. Table 5; Muller classification: Section 5.2.4. Table 6.

**Table 2:** Basic characteristics of VIT.

First Author	Country	Reason	Paper Type	Number of Cases	Venom Type	Skin Test	Injection Amount	Concomitant Treatment	Adverse Events Symptoms	Adverse Events Severity	Adverse Events Type	Mueller Classification	Causality
Castro Neves [79]	Turkey	treatment of systematic allergic reactions	CR	1	bees	tested (positive)	100 μg	NR	none	-	-	-	-
Da Silva [80]	Australia	treatment of systematic allergic reactions	CR	2	bees	tested (positive)	100 μg	NR	(1) none (2) NR	(1) - (2) NR	(1) - (2) NR	(1) - (2) NR	(1) - (2) NR
Ekstrom [81]	Germany	treatment of systematic allergic reactions	CS	A:46 B:68	bees	tested (positive)	NR	omalizumab (4 cases)	NR	NR	NR	NR	NR
Fok [82]	Australia	treatment of systematic allergic reactions	cohort	A:5 B:1	A:bees B:wasp	tested (positive)	100 μgx2	NR	hypotensive systemic reactions	severe	SR	Gr 4	probable
Gür Çetinkaya [83]	Turkey	treatment of systematic allergic reactions	cohort	107	wasp	tested (positive)	NR	NR	local reactions systematic reactions	NR	SP SR	NR	possible
Gür çetinkaya [84]	Turkey	treatment of systematic allergic reactions	CS	107	A:bees B:wasp C:bees,wasp	tested (positive)	NR	NR	NR	NR	SP SR	NR	possible
Kappatou [85]	Greece	treatment of systematic allergic reactions	CR	A:8 B:2	A:wasp B:bees	6 tested (5 positive)	NR	NR	NR	mild	SP SR	NR	possible
Kempinski [86]	Poland	treatment of systematic allergic reactions	CS	246	wasp	NR	NR	NR	field stings anaphylaxis	mild severe	SP SR	Gr1 Gr4	possible probable
Kochuyt [87]	Belgium	treatment of systematic allergic reactions	CS	A:128 B:50	A:wasp B:bees	NR	100 μg	NR	field re-stings	mild	SP SR	Gr1	probable
Kołaczek [88]	Poland	treatment of systematic allergic reactions	cohort	A:34 B:146	A:bees B:wasp	NR	NR	NR	NR	mild	SP SR	Gr1	possible
Mastnik [89]	Germany	treatment of systematic allergic reactions	cohort	A:74 B:124	A:bees B:wasp	NR	A:100~400 μg B:100~200 μg	NR	NR	mild	SR	Gr1	probable
Nittner-Marszalska [90]	Poland	treatment of systematic allergic reactions	cohort	341	bees wasp	NR	NR	NR	NR	mild	SR	Gr1	possible
Puebla Villaescusa [91]	Spain	treatment of systematic allergic reactions	CR	1	bees	NR	40~100 μg	omalizumab (300 mg)	none	-	-	-	-
Rerinck [92]	Germany	treatment of systematic allergic reactions	cohort	A:4 B:21 C:8	A:bees B:wasp C:bees,wasp	NR	100–200 μg	NR	NR	mild	SR	Gr1	possible
Sieber [93]	Germany	treatment of systematic allergic reactions	RCT	A:30 B:30	A:bees B:wasp	NR	~100 mg	NR	anaphylaxis	NR	SP SR	Gr1 Gr4	possible probable
Treudler [94]	Germany	treatment of systematic allergic reactions	CS	20	wasp	NR	~210 mg	NR	NR	NR	NR	NR	NR
Vachová [95]	Czech	treatment of systematic allergic reactions	nRCT	A:80 B:65	A:bees B:wasp	NR	NR	NR	anaphylaxis	mild severe	SP SR	Gr1 Gr4	probable
Vázquez-Revuelta [96]	Spain	treatment of systematic allergic reactions	CR	1	NR	NR	~100 μg	NR	chest tightness oxygen desaturation hypotension	severe	SR	Gr4	probable
Wieczorek [97]	Germany	treatment of systematic allergic reactions	CR	1	wasp	tested	~100 μg	NR	none	-	-	-	-
Arzt-Gradwohl [98]	Australia	treatment of systematic allergic reactions	cohort	1425	bees wasp	NR	NR	NR	NR	NR	NR	NR	NR
Hanzlikova [99]	Czech	treatment of systematic allergic reactions	CR	1	wasp	tested (positive)	NR	cetirizine 10 mg danazol 200 mg	none	-	-	-	-
Lanning [100]	U.S.A.	treatment of systematic allergic reactions	CR	1	wasp	NR	0.1~0.5 mL	NR	rash	mild	SP	Gr1	possible
Nittner-Marszalska [101]	Poland	treatment of systematic allergic reactions	CR	1	wasp	NR	NR	NR	none	-	-	-	-
Pospischil [102]	Australia	treatment of systematic allergic reactions	cohort	A:54 B:93	A:bees B:wasp	NR	NR	NR	cluster rash ultra-rush	NR	SP	Gr1	possible
Toldra [103]	France	treatment of systematic allergic reactions	CR	1	bees	NR	~40 μg	omalizumab (300 mg)	anaphylaxis	severe	SR	Gr4	probable
Goh [104]	Australia	treatment of systematic allergic reactions	cohort	174	bees	NR	NR	NR	NR	NR	NR	NR	NR

VIT: venom immunotherapy

**Table 3:** Basic characteristics of live bee sting.

First Author	Country	Reason	Paper Type	Number of Cases	Venom Type	Skin Test	Injection Amount	Concomitant Treatment	Adverse Events Symptoms	Adverse Events Severity	Adverse Events Type	Mueller Classification	Causality
Utani [105]	Japan	NR	CR	8	bees	NR	NR	-	erythema wheals anaphylaxis	mild severe	SP SR	Gr1 Gr4	probable
Li [106]	China	NR	RCT	A:120 B:120	bees	NR	A: lower B: higher	-	urticaria	mild	SP	Gr1	possible
Wen [107]	China	knee osteoarthritis	CS	43	bees	tested (negative)	NR	Chinese medicine	fever itching urticaria	mild	SP	Gr1	possible
Wen [108]	China	connective tissue disease	CS	40	bees	NR	NR	-	rash mild fever	mild	SP	Gr1	possible
Chen [109]	China	rheumatoid arthritis	RCT	A:60 B:60	A:bees B:-	NR	NR	A:- B: oral methotrexate	none	-	-	-	-
Qin [110]	China	shoulder–hand syndrome after CVA	RCT	A:36 B:36	A:bees B:-	tested (negative)	1~3 point 1~3 ea	A: citicoline 0.75 g, DW5% or NS250 mL, rehabilitation treatment B: acupuncture, citicoline 0.75 g + DW5% or NS250 mL, rehabilitation treatment	none	-	-	-	-
Jiao [111]	China	primary menstrual pain	RCT	A:30 B:30	A:bees B:-	NR	1 ea~4 ea	A:- B: oral ibuprofen capsule	none	-	-	-	-
Wu [112]	China	lumbar disc herniation	RCT	A:40 B:40	A:bees B:	NR	1 ea~10 ea	A: Mckenzie methods, magneto thermal vibration therapy B: Mckenzie methods, magneto thermal vibration therapy	NR	NR	NR	NR	NR

CVA: cerebrovascular accident

**Table 4:** Basic characteristics of external treatments.

First Author	Country	Reason	Paper Type	Number of Cases	Venom Type	Skin Test	Injection Amount	Concomitant Treatment	Adverse Events Symptoms	Adverse Events Severity	Adverse Events Type	Mueller Classification	Causality
Moon [113]	Korea	repigmentation of vitiligo	CR	7	bees	NR	NR	fractional CO_2_ laser	itching erythema persisted hyper pigmentation	mild severe	SP	Gr1	probable
Mo [114]	China	acne	CS	40	bees	NR	NR	-	burning itching desorption dryness	mild	SP	Gr1	possible
Park [115]	Korea	chemotherapy-induced peripheral neuropathy	CR	4	bees	NR	NR	-	none	-	-	-	-
Yang [116]	China	tonsillitis	RCT	A:64B:61	A:beesB:-	NR	NR	A: honey, oral cephaloclonal granules B: oral cephaloclonal granules	NR	NR	NR	NR	NR

**Table 5:** Spilker’s adverse events classification.

Mild	Does Not Significantly Impair Daily Activities (Function) Nor Require Additional Medical Intervention
Moderate	Significantly impairs daily activities (function) and may require additional medical intervention but resolves afterwards
Severe	Serious adverse events that requires intense medical intervention and leaves sequelae

**Table 6:** Classification of systemic reactions to insect stings by Mueller.

Grade Ⅰ	Itch, Urticarial, Malaise, Anxiety
Grade Ⅱ	Any of the above plus two or more of the following: angio-oedema, tight chest, nausea, vomiting, diarrhea, abdominal pain, dizziness
Grade Ⅲ	Any of the above plus two or more of the following: dyspnea, wheeze, stridor, hoarseness, weakness, feeling of impending doom
Grade Ⅳ	Any of the above plus two or more of the following: hypotension, collapse, loss of consciousness, cyanosis

**Table 7:** Characteristics of included RCTs.

Author [Ref]	Condition	Sample Size	Treatment Time	Treatment Period	Intervention	Control	Evaluation Index	Results	*p*-Value (Significance)
Gwo [24]	chronic urticaria	(A) 50 (B) 50	NR	NR	(A) -bee venom injection -herbal medicine	(B) -herbal medicine -acupuncture	(1) Efficacy rate (2) Recurrence rate	(1) (A) 96% (B) 90% (2) (A) 14% (B) 38%	(1) *p* < 0.05 (2) *p* < 0.05
Gwo [25]	ankylosing spondylitis	(A) 30 (B) 30	NR	NR	(A) -bee venom injection -bee’s oral medicine	(B) -western medicine	Efficacy rate	(A) 80.00% (B) 66.67%	significant
Chiu [26]	rheumatoid arthritis	(A) 35 (B)35	NR	NR	(A) -bee venom injection -methotrexine	(B) -methotrexine -prednisolone acetate	(1) Efficacy rate (2) Recurrence rate	(1) (A) 96.49% (B) 65.71% (2) (A) 8.57% (B) 14.29%	(1) *p* < 0.05 (2) *p* > 0.05
Qi [27]	rheumatoid arthritis	(A) 49 (B) 49	NR	NR	(A) -bee venom injection	(B) -western medicine	(1) Efficacy rate (2) Recurrence rate	(1) (A) 95.92% (B) 93.88% (2) (A) 4.08% (B) 16.33%	(1) *p* > 0.05 (2) *p* < 0.05
Su [30]	enlargement of mammary gland	(A) 30 (B) 30 (C) 30	10	NR	(A) -bee venom injection (B) -bee venom injection -acupuncture	(C) -acupuncture	(1) Efficacy rate (2) Breast pain, menstruation (3) Breast mass (4) Emotional changes	(1) (A) 76.67% (B) 93.33% (C) 66.67%	(1) (A),(C) *p* > 0.05 (A),(B) *p* > 0.05 (B),(C) *p* < 0.05 (2) (A),(B),(C) *p* < 0.05 (A),(B) *p* > 0.05(3) (A),(C) *p* < 0.05 (A),(B) *p* > 0.05 (B),(C) *p* < 0.01 (4) (A),(B),(C) *p* > 0.05
An [31]	cancerous pain from lung cancer	(A) 39 (B) 39	NR	20 days	(A) -bee venom injection -hydroxycodone tablets	(B) -hydroxycodone tablets	Efficacy rate	(A) 82.05% (B) 61.54%	*p* < 0.05
Yang [32]	rheumatoid arthritis	(A) 46 (B) 46	NR	30 days	(A) -bee venom injection -herbal medicine	(B) -routine treatment	(1) Efficacy rate (2) Recurrence rate	(1) (A) 97.83% (B) 78.26% (2) (A) 8.70% (B) 28.26%	(1) *p* < 0.05 (2) *p* < 0.05
Wen [33]	ankylosing spondylitis	(A) 40 (B) 40	NR	12 weeks	(A) -bee venom injection	(B) -sulfasalazine -diclofenac	(1) Efficacy rate (2) Adverse events rate	(1) (A) 77.5% (B) 80.0% (2) (A) 7.5% (B) 25%	(1) *p* > 0.05 (2) NR
Wang [34]	cancer pain	(A) 44 (B) 43	NR	NR	(A) -bee venom injection -fentanyl percutaneous patch	(B) -fentanyl percutaneous patch	(1) Efficacy rate (2) Quality of life (3) Pain intensity (4) Adverse event rate	NR	(1) *p* < 0.01 (2) *p* < 0.05 (3) *p* < 0.05 (4) *p* < 0.01
Zhang [35]	frozen shoulder	(A) 33 (B) 32 (C) 32	NR	NR	(A) -bee venom injection -acupotomy	(B) -acupotomy -triamcinolone acetonide (C) -acupotomy	Efficacy rate	(A) 100% (B) 100% (C) 93.75%	NR
Zhang [36]	facial palsy	(A) 36 (B) 35	NR	4 weeks	(A) -bee venom injection -acupuncture	(B) -acupuncture	(1) H-B Grade (2) Sunnybrook scale (3) Efficacy rate	(1) NR (2)NR (3) (A) 97.1% (B) 89.9%	(1) *p* > 0.05 (2) *p* < 0.05 (3) *p* < 0.05
Zhou [38]	rheumatoid arthritis	(A) 40 (B) 30 (C) 30	NR	NR	(A) -bee venom injection	(B) -electro acupuncture (C) -western medicine	Blood test level	NR	NR
Zeng [40]	ankylosing spondylitis	(A) 54 (B) 54	NR	NR	(A) -bee venom injection -moxibustion	(B) -acupuncture	Efficacy rate	(A) 74.07% (B) 42.31%	*p* < 0.05
Chen [42]	rheumatoid arthritis	(A) 30 (B) 30	NR	NR	(A) -bee venom injection	(B) -oral methotrexate -celecoxib	(1) Efficacy rate (2) VAS	NR	(1) *p* < 0.05 (2) *p* < 0.05
Peng [43]	cancer pain	(A) 31 (B) 33	NR	NR	(A) -bee venom injection -tramadol 100 mg	(B) -tramadol 100 mg	(1) Pain relief (2) Quality of life (3) Adverse event relief (4) Systemic symptoms	NR	(1) *p* < 0.01 (2) *p* > 0.05 (3) *p* < 0.05 (4) *p* < 0.05
Peng [44]	cancer pain	(A) 30 (B) 30	30	30 days	(A) -bee venom injection -pain medicine 3rd phase (WHO recommended)	(B) -pain medicine 3rd phase (WHO recommended)	(1) Efficacy rate (2) Adverse event rate	(1) (A) 96.67% (B) 90.00% (2)NR	(1) *p* < 0.05 (2) *p* < 0.05
Huang [45]	rheumatoid arthritis	(A) 30 (B) 30	30	NR	(A) -bee venom injection	(B) -hemp tablets	Efficacy rate	(A) 100% (B) 86.7%	*p* < 0.01
Guo [46]	ankylosing spondylitis	(A) 36 (B) 36	NR	NR	(A) -bee venom injection	(B) -western treatment	Efficacy rate	(A) 94.44% (B) 72.22%	significant
Deng [47]	rheumatoid arthritis	(A) 20 (B) 20 (C) 20	NR	60 days	(A) -bee venom injection -metrotrexate	(B) -metrotrexate (C) -strong metrotrexate	(1) Clinical symptoms (2) Blood test level	NR	NR
Liu [48]	rheumatoid arthritis	(A) 50 (B) 50	NR	3 months	(A) -bee venom injection -western medicine	(B) -western medicine	(1) Efficacy rate (2) Symptoms (3) Adverse event and recurrence rate	NR	(1) *p* < 0.05 (2) *p* < 0.05 (3) *p* < 0.05
Yang [49]	diabetic neuropathy	(A) 25 (B) 25	15	15 days	(A) -bee venom injection -epalrestat -methylcobalamin	(B) -epalrestat -methylcobalamin	(1) Neurotransmission speed (2) Hydrogen peroxide enzyme level (3) Glutathione level	NR	(1),(2),(3) *p* < 0.05
Wen [50]	postpartum pain	(A) 41 (B) 40	NR	8 weeks	(A) -bee venom injection -herbal fumigation	(B) -diclofenac natrium minidose	Efficacy rate	(A) 95.2% (B) 77.5%	*p* < 0.01
Wen [51]	postherpetic neuralgia	(A) 36 (B) 36	NR	12 weeks	(A) -bee venom injection	(B) -injection(unknown)	(1) Efficacy rate (2) Blood serum test (3) Adverse event rate	(1) (A) 97.22% (B) 77.78% (2),(3) NR	(1) *p* < 0.05 (2) *p* < 0.05 (3) *p* > 0.05
Wei [52]	rheumatoid arthritis	(A) 30 (B) 30	NR	NR	(A) -bee venom injection	(B) -Chinese medicine	(1) Efficacy rate (2) VAS (3) Blood serum test (4) Adverse event rate	(1) (A) 90.00% (B) 66.66% (2),(3),(4) NR	(1) *p* < 0.05 (2) *p* > 0.05 (3) *p* < 0.05 (4) *p* > 0.05
Ying [53]	Shoulder pain	(A) 60 (B) 60	NR	4 weeks	(A) -bee venom injection	(B) -massage -acupuncture	(1) Efficacy rate (2) McGill pain scale (3) Constant-Murley score (4) Ridiet analysis	(1) (A) 95.00% (B) 81.67% (2),(3),(4) NR	(1) *p* < 0.05 (2) *p* < 0.01 (3) *p* < 0.01 (4) *p* < 0.05
Zhou [54]	neurotic tinnitus	(A) 30 (B) 30	NR	4 weeks	(A) -bee venom injection -heating needle	(B) -flunarizine hydrochloride capsule -mecobalamin minidose	(1) Hearing impairment threshold level (2) Tinnitus (3) SDS level (4) Efficacy rate	(1),(2),(3) NR (4) (A) 83.33% (B) 66.67%	(1) *p* < 0.01 (2) *p* < 0.01 (3) *p* < 0.01 (4) *p* < 0.05
Chen [56]	Rheumatoid arthritis	(A) 30 (B) 30 (C) 30	(A),(B) 24 (C) 56	8 weeks	(A) -bee venom injection (high dose) (B) -bee venom injection (low dose)	(C) -methotrexate 10 mg -cerecoxib 0.2 g	Efficacy rate	(A) 86.67% (B) 70.00% (C) 76.67	*p* < 0.05
Qin [57]	rheumatoid arthritis	(A) 32 (B) 28	NR	3 months	(A) -bee venom injection -xianlong granule	(B) -methotrexate	(1) Efficacy rate (2) TCM syndrome score (3) VAS (4) DAS (5) HAQ score (6) Adverse event rate	(1) (A) 90.6% (B) 85.7% (2),(3),(4),(5),(6) NR	(1) *p* > 0.05 (2) *p* > 0.05 (3) *p* > 0.05 (4) *p* > 0.05 (5) *p* < 0.05
Won [63]	osteoarthritis	(A) 25 (B) 26 (C) 26 (D) 24	42	6 weeks	(A) -bee venom injection(~0.7 mg) (B) -bee venom injection(~1.5 mg) (C) -bee venom injection(~2.0 mg)	(D) -nabumetone 1000 mg	Efficacy rate	NR	(A),(B),(C):(D) *p* < 0.01 (B),(C):(A) *p* < 0.01
Lee [71]	non-specific neck pain	(A) 30 (B) 30	NR	≥ 3 months	(A) -bee venom injection	(B) -loxoprofen 180 mg	Clinical symptoms	NR	NR
Chen [109]	rheumatoid arthritis	(A) 60 (B) 60	(A) 24 (B) 56	8 weeks	(A) -live bee sting	(B) -oral methotrexate	(1) Efficacy rate (2) Morning stiffness, joint pain/edema/tenderness index, grip strength, 15 min walking time, VAS, rheumatoid factor, CRP level	(1) (A) 83.33% (B) 80.00% (2) NR	(1) *p* > 0.05 (2) *p* > 0.05
Qin [110]	shoulder–hand syndrome after CVA	(A) 36 (B) 36	live bee sting 9 acupuncture 18 rehabilitation 18 fluid 21	3 weeks	(A) -live bee sting -citicoline 0.75 g -DW5% or NS250 mL -rehabilitation treatment	(B) -acupuncture -citicoline 0.75 g -DW5% or NS250 mL -rehabilitation treatment	(1) Efficacy rate (2) VAS	(1) (A) 93.75% (B) 73.53% (2) NR	(1) *p* < 0.05 (2) *p* < 0.01
Jiao [111]	primary menstrual pain	(A) 30 (B) 30	(A) 10 (B) NR	3 months	(A) -live bee sting	(B) -oral ibuprofen capsules	(1) Efficacy rate (2) Adverse event rate	(1) (A) 93.3% (B) 76.6% (2) (A) 100% (B) 0%	(1) *p* < 0.05 (2) *p* < 0.05
Wu [112]	lumbar disc herniation	(A) 40 (B) 40	live bee sting, magneto thermal vibration therapy 14 Mckenzie methods 7	2 weeks	(A) -live bee sting -Mckenzie methods -Magneto thermal vibration therapy	(B) -Mckenzie methods -Magneto thermal vibration therapy	(1) VAS (2) ODI (3) TCM score (4) Clinical efficacy rate	(1),(2),(3) NR (4) (A) 95% (B) 80%	(1) *p* < 0.05 (2) *p* > 0.05 (3) *p* < 0.05 (4) *p* < 0.05
Yang [116]	Tonsillitis	(A) 64 (B) 61	10	5 days	(A) -bee venom externals -honey externals -oral cephaloclonal granules	(B) -oral cephaloclonal granules	(1) Efficacy rate (2) Adverse event rate	(1) (A) 100% (B) 90.2% (2) (A) 3.1% (B) 1.6%	(1) *p* < 0.05 (2) *p* > 0.05

NR: not reported; H-B grade: House–Brackmann grade; VAS: Visual Analog Scale; SDS: Self-Depression Scale; TCM syndrome score: Traditional Chinese Medicine syndrome score; DAS: Diseases Activity Score; HAQ: Health Assessment Questionnaire; CRP: C-reactive protein; ODI: Oswestry Low Back Pain Disability Index.

**Table 8:** WHO-UMC causality categories.

Causality Term	Assessment Criteria
Certain	-Event or laboratory test abnormality, with plausible time relationship to drug intake -Cannot be explained by disease or other drugs -Response to withdrawal plausible (pharmacologically, pathologically) -Event definitive pharmacologically or phenomenologically (i.e., and objective and specific medical disorder or a recognized pharmacological phenomenon) -Rechallenge satisfactory, if necessary
Probable/ Likely	-Event or laboratory test abnormality, with reasonable time relationship to drug intake -Unlikely to be attributed to disease or other drugs -Response to withdrawal clinically reasonable -Rechallenge not required
Possible	-Event or laboratory test abnormality, with reasonable time relationship to drug intake -Could also be explained by disease or other drugs -Information on drug withdrawal may be lacking or unclear
Unlikely	-Event or laboratory test abnormality, with a time to drug intake that makes a relationship improbable (but not impossible) -Disease or other drugs provide plausible explanations
Conditional/ Unclassified	-Event of laboratory test abnormality -More data for proper assessment needed, or -Additional data under examination
Unassessable/ Unclassifiable	-Report suggesting and adverse reaction -Cannot be judged because information is insufficient or contradictory -Data cannot be supplemented or verified

## Data Availability

The data used in this study are available from the corresponding author upon request.
